# Voltage-Gated Sodium Channel β1/β1B Subunits Regulate Cardiac Physiology and Pathophysiology

**DOI:** 10.3389/fphys.2018.00351

**Published:** 2018-04-23

**Authors:** Nnamdi Edokobi, Lori L. Isom

**Affiliations:** Department of Pharmacology, University of Michigan Medical School, Ann Arbor, MI, United States

**Keywords:** sodium channel, B subunit, arrhythmia, epilepsy, cell adhesion, electrophysiology

## Abstract

Cardiac myocyte contraction is initiated by a set of intricately orchestrated electrical impulses, collectively known as action potentials (APs). Voltage-gated sodium channels (Na_V_s) are responsible for the upstroke and propagation of APs in excitable cells, including cardiomyocytes. Na_V_s consist of a single, pore-forming α subunit and two different β subunits. The β subunits are multifunctional cell adhesion molecules and channel modulators that have cell type and subcellular domain specific functional effects. Variants in *SCN1B*, the gene encoding the Na_v_-β1 and -β1B subunits, are linked to atrial and ventricular arrhythmias, e.g., Brugada syndrome, as well as to the early infantile epileptic encephalopathy Dravet syndrome, all of which put patients at risk for sudden death. Evidence over the past two decades has demonstrated that Na_v_-β1/β1B subunits play critical roles in cardiac myocyte physiology, in which they regulate tetrodotoxin-resistant and -sensitive sodium currents, potassium currents, and calcium handling, and that Na_v_-β1/β1B subunit dysfunction generates substrates for arrhythmias. This review will highlight the role of Na_v_-β1/β1B subunits in cardiac physiology and pathophysiology.

## Introduction

**Figure 1:**
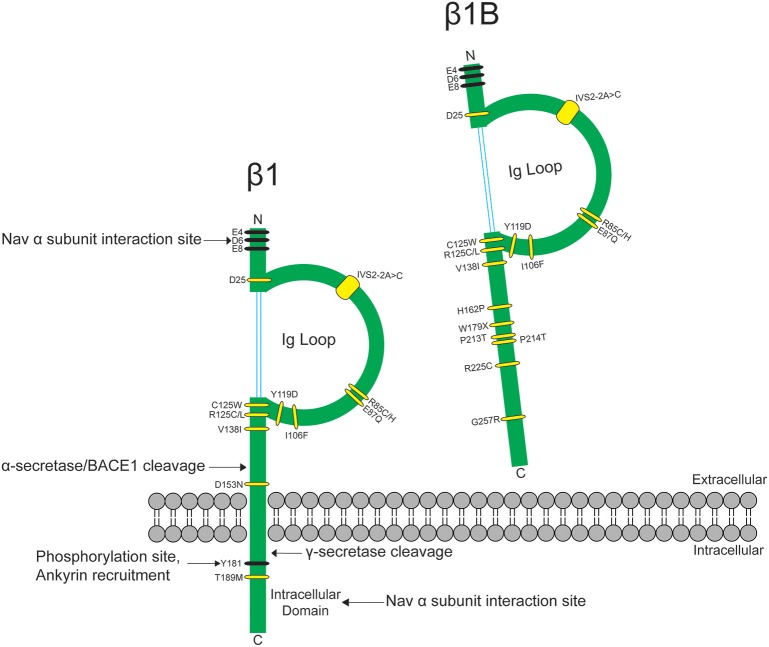
*SCN1B* variants are linked to epilepsy syndromes and cardiac conduction diseases. *SCN1B* encodes Na_v_-β1 (**left**) and its secreted splice variant Na_v_-β1B (**right**). Sites for β1-α interaction, ankyrin binding, phosphorylation, and proteolytic cleavage are indicated. Ig: immunoglobulin. Human disease variants in β1 or β1B are indicated in yellow and are described in Table 1. Adapted from O'Malley and Isom (2015).

The heart contracts to pump blood throughout the body. It consists of specialized cells called cardiac myocytes (CMs), and contraction of CMs is initiated by electrical impulses called action potentials (APs) (Nerbonne and Kass, 2005). Cardiac APs are generated and propagated through the coordinated signaling of ion channels. Upon membrane depolarization, voltage-gated sodium channels (Na_V_s) activate and inactivate rapidly to allow sodium influx (Hille and Catterall, 2012). This is responsible for the rising phase and propagation of the AP in mammalian CMs (Nerbonne and Kass, 2005). Na_V_s are heterotrimeric transmembrane proteins consisting of one pore-forming α and two β subunits (Catterall, 2000). Na_V_-β subunits are expressed in mammalian heart (Isom et al., 1992; Makita et al., 1994) and their functional loss can result in electrical abnormalities that predispose patients to arrhythmias. Variants in the gene *SCN1B*, encoding the splice variants Na_V_-β1 and Na_V_-β1B, are implicated in a variety of inherited pathologies including epileptic encephalopathy (O'Malley and Isom, 2015), Brugada syndrome (BrS) (Watanabe et al., 2008; Hu et al., 2012), long-QT syndrome (LQTS) (Riuró et al., 2014), atrial arrhythmias (Watanabe et al., 2009), and sudden infant death syndrome (SIDS) (Hu et al., 2012) (Figure 1, Table 1). Remarkably, regardless of disease etiology, patients with *SCN1B* mutations have an increased risk of sudden death. Classically, the Na_V_-β subunits were characterized as modulators of the Na_V_ ion-conducting pore. However, from research over the past two decades, we know that Na_V_-β subunits are dynamic, multifunctional proteins that play important roles in cardiac physiology (O'Malley and Isom, 2015). Here, we will focus our review on the current understanding of Na_V_-β1/β1B function in CMs and discuss disease implications.

**Table 1:** *SCN1B* variants linked to human disease.

**Disease**	**β1**	**β1B**
Atrial fibrillation	R85H (Watanabe et al., 2009), D153N (Watanabe et al., 2009)	R85H (Watanabe et al., 2009), D153N (Watanabe et al., 2009)
Brugada syndrome	E87Q (Watanabe et al., 2008)	E87Q (Watanabe et al., 2008), H162P (Holst et al., 2012), W179X, R214Q (Holst et al., 2012; Hu et al., 2012)
Dravet syndrome	I106F (Ogiwara et al., 2012), Y119D (Ramadan et al., 2017), C121W (Wallace et al., 1998), R125C (Patino et al., 2009)	I106F (Ogiwara et al., 2012), Y119D (Ramadan et al., 2017),C121W (Wallace et al., 1998), R125C (Patino et al., 2009)
Generalized Epilepsy with Febrile Seizures Plus (GEFS+)	D25N (Orrico et al., 2009), R85H (Scheffer et al., 2006), R85C (Scheffer et al., 2006), R125L (Fendri-Kriaa et al., 2011), five amino acid deletions (IVS2-2A>C) (Audenaert et al., 2003)	D25N (Orrico et al., 2009), R85H (Scheffer et al., 2006), R85C (Scheffer et al., 2006), R125L (Fendri-Kriaa et al., 2011), five amino acid deletions (IVS2-2A>C) (Audenaert et al., 2003)
Idiopathic epilepsy		G257R (Patino et al., 2011)
Sudden Infant Death Syndrome (SIDS)		R214Q (Hu et al., 2012), R225C (Neubauer et al., in press)
Sudden Unexpected Nocturnal Death Syndrome (SUNDS)	V138I (Liu et al., 2014), T189M (Liu et al., 2014)	V138I (Liu et al., 2014)
Long QT Syndrome (LQTS)		P213T (Riuró et al., 2014)

## Na_V_s are differentially expressed in cardiac myocytes

**Figure 2:**
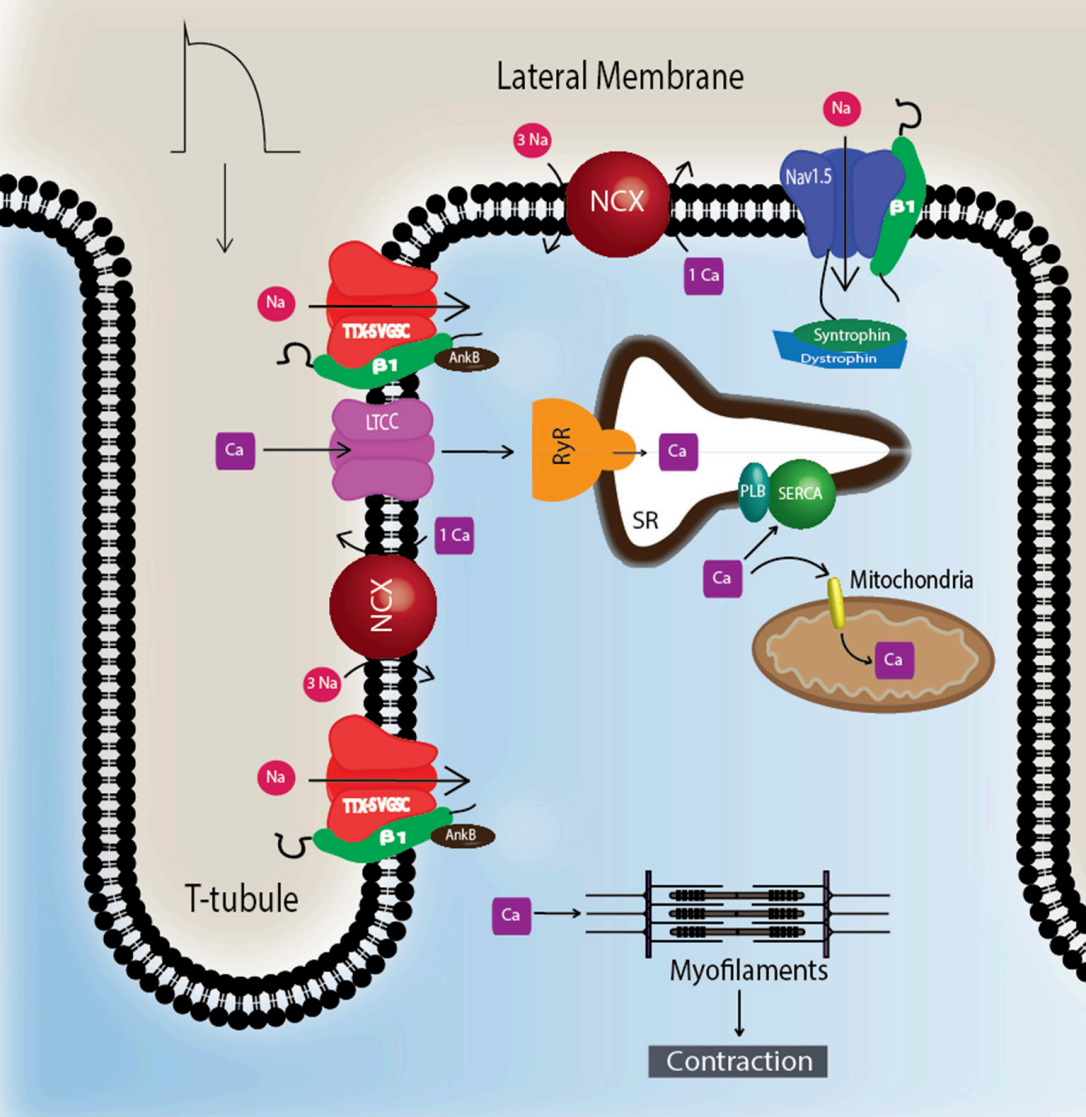
TTX-S Na_v_s are localized to T-tubules. TTX-S Na_v_s, including Na_v_1.1, Na_v_1.3, and Na_v_1.6, are located at the T-tubules of CMs where they are thought to participate in the regulation of excitation-contraction coupling. Non-phosphorylated Na_v_-β1 subunits are co-localized with TTX-S Na_v_-α subunits at the T-tubules where they play roles in calcium signaling and homeostasis. Na_v_1.5 is localized at the lateral membrane as well as the ID (Figure 3). At the lateral membrane, Na_v_1.5 is complexed with syntrophin and dystrophin. Abbreviations: L-type calcium channel (LTCC), phospholamban (PLB), ryanodine receptor (RyR), sarcoplasmic reticulum Ca^2+^-ATPase (SERCA), sodium-calcium exchanger (NCX), transverse tubules (T-tubule).

To understand Na_v_-β subunit physiology in heart, one must first consider the associated Na_v_-α subunits. Na_v_1.5 is the predominantly expressed Na_V_-α in CMs and the primary contributor to recorded sodium current (I_Na_) density (Rogart et al., 1989; Gellens et al., 1992; Catterall, 2000; Maier et al., 2002). Na_v_1.5 is a “tetrodotoxin resistant (TTX-R)” channel (Catterall et al., 2005), in contrast to “TTX-sensitive (TTX-S)” channels, e.g., Na_V_s normally found in brain, for which TTX has nanomolar affinity (Catterall et al., 2005). TTX has micromolar affinity for Na_v_1.5 due to the presence of a cysteine residue in the selectivity filter in a position that is otherwise filled by an aromatic amino acid in TTX-S channels (Satin et al., 1992). TTX-S channels, Na_v_1.1, Na_v_1.3, and Na_v_1.6, are expressed in heart as well as in brain (Malhotra et al., 2001; Lopez-Santiago et al., 2007). They are preferentially localized in the transverse tubules (T-tubules) (Malhotra et al., 2001, 2002; Lopez-Santiago et al., 2007) where they are postulated to function in excitation-contraction coupling (Maier et al., 2002) (Figure 2).

**Figure 3:**
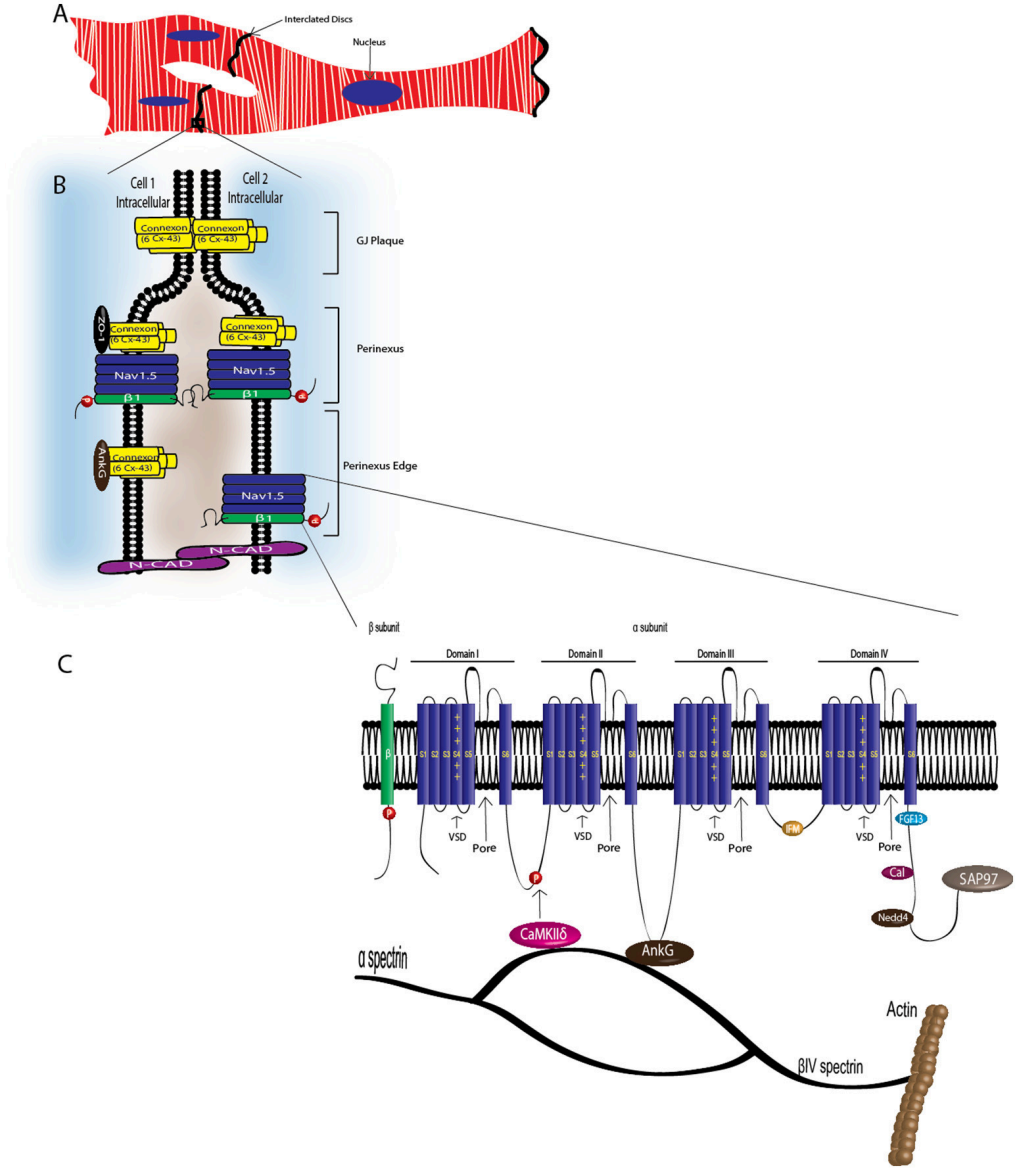
VGSC complexes at the cardiac intercalated disk. CMs associate at the ID, where Na_v_1.5, Nav-β1 subunits, adherens junctions, gap junctions, and desmosomes define intercellular communication. **(A)** Associated CMs. **(B)** Proposed model of the GJ plaque, perinexus, and perinexus edge. Nav-β1 subunits at the ID are tyrosine phosphorylated, possibly through Fyn kinase activation, and may function in cell–cell adhesion in the perinexus and perinexus edge. **(C)** At the ID, Nav1.5 associates with a multi-protein complex (also see Table 2). The S4 segment of each Na_v_-α subunit homologous domain forms the voltage sensing domain (VSD) and segments 5 and 6 in each domain create the ion-conducting pore. Three hydrophobic amino acids, IFM, form the inactivation gate. Abbreviations: Ankyrin-G (AnkG), Calmodulin (Cal), Ca^2+^/calmodulin-dependent protein kinase II (CaMKIIδ), Fibroblast growth factor homologous factor 1B (FHF1B), N-Cadherin (N-Cad), Nedd4-like-ubiqutin-protein ligases (Nedd4), Synapse-associated protein 97 (SAP97).

CMs associate at the intercalated disk (ID), where adherens junctions, gap junctions, and desmosomes participate in intercellular communication (Vermij et al., 2017) (Figure 3A). Na_v_1.5 channels cluster at cell–cell junction sites at the ID, where they co-localize with the cardiac gap junction (GJ) protein, connexin-43 (Cx43) (Maier et al., 2002, 2004) (Figure 3B). Na_v_1.5 clustering may contribute to rapid AP conduction from cell-to-cell, similar to the node-to-node saltatory conducting function of TTX-S Na_V_s in myelinated nerves (Freeman et al., 2016). Na_v_1.5 channels are also expressed at the CM lateral membrane (Figure 2), where they have differing biophysical properties and binding partners from those at the ID (Lin et al., 2011; Petitprez et al., 2011; Shy et al., 2013), suggesting two distinct Na_v_1.5 pools.

## Cardiac Na_V_s form multi-protein complexes

Na_V_-α subunits interact with multi-protein complexes that are subcellular domain specific in heart. These interactions, which involve Na_v_-β1, as discussed throughout this review, are essential for proper cardiac electrical signaling (Figure 3C, Table 2). Ankyrin-G, a cytoskeletal adaptor protein, is necessary for normal expression of Na_v_1.5 and coupling of the channel to the actin cytoskeleton (Mohler et al., 2004). A human *SCN5A* BrS variant eliminates Na_v_1.5-ankyrin-G interactions (Mohler et al., 2004). This mutation, located in the Na_v_1.5 DII–III loop, prevents channel cell surface expression in ventricular CMs and alters channel properties. In agreement with this result, rat CMs with reduced expression of ankyrin-G have reduced levels of Na_v_1.5 expression and I_Na_. Abnormal Na_v_1.5 localization can be rescued in ankyrin-G deficient CMs through exogenous over-expression of ankyrin-G (Lowe et al., 2008). Ankyrin-G recruits βIV spectrin, which forms important scaffolding structures and plays a role in the maintenance and integrity of the plasma membrane and cytoskeleton (Yang et al., 2007). βIV spectrin associates with and targets a subpopulation of Ca^2+^/calmodulin-dependent protein kinase II (CaMKIIδ) to the ID to phosphorylate a critical serine residue in the Na_v_1.5 I–II linker (Hund et al., 2010; Makara et al., 2014). Mouse CMs expressing a mutant form of βIV spectrin show a positive shift in I_Na_ steady-state inactivation, elimination of late I_Na_, shortened APD, and decreased QT intervals (Hund et al., 2010), confirming that formation of the Na_v_1.5-ankyrin-G signaling complex is critical for maintaining normal cardiac excitability.

**Table 2:** Na_v_1.5 ID interacting proteins.

**Interacting protein**	**Effects on Na_v_1.5**	**Reference(s)**
Ankyrin-G (AnkG)	Proper expression at plasma membrane and coupling to actin cytoskeleton	Mohler et al., 2004
Calmodulin (Cal)	Regulates biophysical properties	Tan et al., 2002; Kim et al., 2004; Young and Caldwell, 2005; Gabelli et al., 2014
Ca^2+^/calmodulin-dependent protein kinase II (CaMKIIδ)	Phosphorylation and modulates excitability	Hund et al., 2010; Makara et al., 2014
Fibroblast growth factor homologous factor 1B (FHF1B)	Modulate channel gating	Liu et al., 2003
Nedd4-like-ubiqutin-protein ligases (Nedd4)	Ubiquitination and regulated internalization. Possible mechanism in modulation of channel density at the plasma membrane	Van Bemmelen et al., 2004; Rougier et al., 2005
Synapse-associated protein 97 (SAP97)	Stability and anchoring to the cell membrane	Petitprez et al., 2011; Matsumoto et al., 2012

Cytoskeletal integrity is a pre-requisite for normal electrical coupling. During cardiac development, GJ proteins and Na_v_1.5 appear at the ID after formation of adherens junctions (Vreeker et al., 2014). The perinexus, a newly identified region of the ID, is defined as the area surrounding the plaque of functional GJs (Rhett et al., 2013) (Figure 3B). Here, free connexons appear at the periphery of the GJ, after which they bind to zonula occludens1 (ZO-1). GJs form when ZO-1 free connexons from one cell associate with ZO-1 free connexons of a neighboring cell (Rhett et al., 2011). Disruption of Cx43/ZO-1 interactions increases GJ size (Hunter, 2005), and in a ZO-1 null model, GJ plaques are larger (Palatinus et al., 2010). Cx43 also interacts with Na_v_1.5 in the perinexus (Rhett et al., 2012). The presence of Na_v_1.5 at the perinexus may suggest that, in addition to GJ proteins, Na_V_s may participate in coupling across the extracellular space, with increasing evidence supporting that both Cx43 and Na_v_1.5 are necessary for cell-to-cell transmission of APs (Gutstein et al., 2001; Lin et al., 2011; Jansen et al., 2012).

Na_v_1.5 contributes to at least two distinct multiprotein complexes in ventricular CMs, one at the lateral membrane containing dystrophin and syntrophin (Figure 2), and the other at the ID involving the membrane-associated guanylate kinase (MAGUK) protein adapter protein, synapse-associated protein 97 (SAP97), and ankyrin-G (Petitprez et al., 2011) (Figure 3C). In heterologous cells, surface expression of Na_v_1.5 is regulated by its interaction with SAP97 via a PDZ-domain (post-synaptic density protein-PSD95, disc large tumor suppressor-Dlg1, zonula occludens1-ZO1). Either the truncation of the fourth domain of Na_v_1.5 (Shy et al., 2014) or depletion of SAP97 (Matsumoto et al., 2012) results in reduced channel cell surface expression, with a subsequent decrease of I_Na_.

Na_v_1.5 also interacts with fibroblast growth factor homologous factor 1B (FHF1B) (Liu et al., 2003), calmodulin (Kim et al., 2004; Young and Caldwell, 2005), Nedd4-like-ubiqutin-protein ligases (Van Bemmelen et al., 2004; Rougier et al., 2005), and is phosphorylated by Fyn (Ahern et al., 2005), a src family tyrosine kinase, all of which are involved in the regulation of channel subcellular localization and activity (Figure 3C). Taken together, these results accentuate the idea that cardiac Na_V_s associate with protein complexes that are specific to subcellular domains, and these interactions are critical to cardiac physiology. Undoubtedly, changes in one component of a given complex results in significant consequences to overall cardiac excitability and synchrony.

## Na_V_-β subunits modulate cardiac excitability

In mammalian genomes, five Na_V_-β subunits are encoded by four genes, *SCN1B-SCN4B* (O'Malley and Isom, 2015). Na_V_-β1-β4 are transmembrane proteins with type 1 topology consisting of an extracellular N-terminus containing an immunoglobulin (Ig) domain, a transmembrane segment, and an intracellular C-terminus (Brackenbury and Isom, 2011) (Figure 1). Na_V_-β1B, a splice variant of *SCN1B*, contains the Na_V_-β1 N-terminal and Ig domains, but lacks a transmembrane domain (Kazen-Gillespie et al., 2000), resulting in a secreted protein (Patino et al., 2011) (Figure 1). Na_V_-β subunits can interact both covalently and non-covalently with Na_V_-α subunits: Na_V_-β1 and -β3 interact non-covalently with Na_V_-α via their N- and C-termini (McCormick et al., 1998; Meadows et al., 2001), while Na_V_-β2 and -β4 interact covalently with Na_V_-α via a single N-terminal cysteine located in the extracellular Ig loop (Chen et al., 2012; Gilchrist et al., 2013).

Canonically, Na_V_-βs are known as modulators of Na_V_ electrophysiological properties and cell surface expression (Brackenbury and Isom, 2011). Heterologous expression systems and mouse models have shown that Na_V_-βs modulate Na_V_−αs in cell type specific manners, thus the Na_V_ α/β subunit composition of a given cell confers unique biophysical properties that can be finely tuned (Calhoun and Isom, 2014). Not surprisingly, Na_V_-β1 modulation of Na_v_1.5 varies depending on the system studied. In *Xenopus* oocytes, the amplitude of Na_v_1.5 expressed I_Na_ increases with increasing amounts of β1 mRNA (Qu et al., 1995). Antisense-mediated post-transcriptional silencing of *Scn1b* in H9C2, a CM line, alters TTX-S and TTX-R Na_v_-α mRNA and protein expression, resulting in decreased I_Na_ (Baroni et al., 2014). In contrast, *Scn1b* null mouse CMs have increased expression of *Scn3a* and *Scn5a*, along with increased TTX-S and TTX-R I_Na_ (Lopez-Santiago et al., 2007). In heterologous cells, Na_V_-β1 expression results in slight changes in Na_v_1.5 I_Na_, but significant effects on voltage-dependence and channel kinetics. In Tsa201 cells transfected with Na_v_1.5, co-expression of Na_V_-β1 positively shifts the voltage-dependence of inactivation (Malhotra et al., 2001). Co-expression of Na_V_-β1 with Na_v_1.5 in *Xenopus* oocytes causes a depolarizing shift in steady-state inactivation compared with WT alone (Zhu et al., 2017), suggesting that β1 may allow the α subunit voltage-sensing domains to recover more rapidly to the resting state. Thus, Na_V_-β1 may initiate fine-tuned acute and chronic feedback mechanisms that differentially control expression and function of Na_V_-αs in the heart.

Na_v_-β1B is expressed in fetal brain and in heart at all developmental time points. When expressed alone or in the presence of TTX-S Na_v_-αs in a heterologous expression system, Na_v_-β1B is secreted (Patino et al., 2011). Secreted Na_v_-β1B functions as a CAM ligand to promote signal transduction in cultured neurons (Patino et al., 2011). In contrast, Na_V_-β1B is retained at the cell surface when co-expressed with Na_v_1.5 (Patino et al., 2011) and Na_v_-β1B co-expression increases I_Na_ density compared to Na_V_1.5 alone (Watanabe et al., 2008). The disease variant, β1B-G257R (Figure 1, Table 1), causes Na_v_-β1B to be retained inside the cell, resulting in a functional null phenotype (Patino et al., 2011). The variant, β1B-W179X (Figure 1, Table 1), fails to increase Na_v_1.5 I_Na_ density, suggesting that it may also be a functional null mutation (Watanabe et al., 2008). A number of Na_v_-β1B variants have now been linked to cardiac arrhythmias (Figure 1, Table 1), thus this subunit is critical to cardiac physiology.

While the Na_v_-αs are known to form and function as monomers, recent evidence suggests that they can also form dimers, and that dimerization is mediated through an interaction site within the first intracellular loop (Clatot et al., 2017). Na_V_-α dimers display coupled gating properties, which are mediated through the action of 14-3-3 proteins (Clatot et al., 2017). The 14-3-3 family of proteins is important for the regulation of cardiac I_Na_, and disrupted 14-3-3 expression may exert pro-arrhythmic effects on cardiac electrical properties (Allouis et al., 2006; Sreedhar et al., 2016). The functional importance of cardiac Na_V_-α dimerization may be to target and enhance the density of channels at specific subcellular domains. Na_v_1.5-R1432G, a surface localization defective *SCN5A* mutant, displays a dominant negative effect on WT Na_v_1.5, but only in the presence of Na_V_-β1 (Mercier et al., 2012). Thus, Na_V_-β1 may normally mediate physical interactions between Na_v_1.5 dimers, however further research must be performed.

## Na_V_-βs do more than modulate I_NA_

Na_V_-β subunits are multifunctional (O'Malley and Isom, 2015). In addition to modulating channel gating and cell surface expression/localization, Na_V_-βs are Ig superfamily cell adhesion molecules (CAMs) that facilitate cell–cell communication and initiate intracellular signaling cascades. Na_V_-β1 and -β2 participate in *trans*-homophilic cell adhesion, resulting in the recruitment of ankyrin-G to the plasma membrane at sites of cell–cell contact (Malhotra et al., 2000). Importantly, this occurs both in the presence and absence of Na_V_-α, at least *in vitro*. Na_V_-β1 and -β2 also participate in cell–matrix adhesion, binding tenascin-R and tenascin- C to modulate cell migration (Srinivasan et al., 1998; Xiao et al., 1999). The Na_V_-β3 amino acid sequence is most similar to Na_V_-β1 compared to the other Na_V_-β subunits (Morgan et al., 2000). While Na_V_-β3 does not function as a CAM when expressed in *Drosophila* S2 cells, as shown for Na_V_-β1 and -β2 (Chen et al., 2012), it does so in mammalian cells where *trans* homophilic adhesion was shown to require an intact Cys2–Cys24 disulfide bond (Yereddi et al., 2013).

Na_V_-β function, localization, and expression are regulated by multiple post-translational modifications including phosphorylation, glycosylation, and proteolytic cleavage (Calhoun and Isom, 2014). All Na_V_-βs have highly glycosylated N-terminal domains, containing 3 to 4 N-linked glycosylation sites each (Isom et al., 1992; McCormick et al., 1998; Johnson et al., 2004), and these modifications contribute to cell surface expression and channel modulation (Johnson et al., 2004). Lastly, Na_V_-βs are targets for sequential proteolytic cleavage by α-secretase/BACE1 and γ-secretase, resulting in the release of N-terminal and C-terminal domains (Wong et al., 2005). These cleavage products may have important physiological effects on transcriptional regulation of Na_V_-α subunit genes. For example, γ-secretase cleavage of Na_V_-β2 in neurons *in vitro* leads to translocation of the intracellular domain to the nucleus, where it increases *SCN1A* mRNA expression and Na_v_1.1 protein (Kim et al., 2007).

Of the five Na_v_-β subunits, Na_v_-β1 has been the most studied in terms of its CAM function. In the heart, Na_V_-β1 ID localization suggests a role in cardiac cell–cell contact. *Scn1b* and *Scn5a* have overlapping temporal and spatial expression profiles during heart development (Domínguez et al., 2005). In ventricular CMs, Na_V_-β1 is co-localized at the ID (Kaufmann et al., 2010) with Na_v_1.5 (Maier et al., 2004), as well as at the T-tubules with TTX-S channels (Malhotra et al., 2001; Lopez-Santiago et al., 2007). Recent evidence suggests that Na_V_-β1-mediated cell–cell adhesion may occur at the perinexal membrane, and this putative interaction can be acutely inhibited by βadp1, a novel peptide mimetic of the Na_V_-β1 CAM domain (Veeraraghavan et al., 2016). Dose-dependent administration of βadp1 decreased cellular adhesion in Na_V_-β1-overexpressing fibroblasts. 75% of βadp1-treated hearts exhibited spontaneous ventricular tachycardias, revealing preferential slowing of transverse conduction. These data support a role for *trans* Na_V_-β1-mediated cell–cell adhesion at the perinexal membrane and suggest a role for adhesion in conduction (Figure 3B). Because a large proportion of *SCN1B* disease variants affect the Ig domain (Figure 1), it is likely that disruption of Na_V_-β1-mediated cell–cell adhesion contributes to disease mechanisms and, if so, that restoring adhesion may be a future therapeutic target.

The Na_V_-β1 intracellular domain can be phosphorylated at tyrosine (Y) residue 181 (Malhotra et al., 2002, 2004; McEwen et al., 2004), possibly through activation of Fyn kinase (Brackenbury et al., 2008; Nelson et al., 2014) (Figure 1). β1Y181E, a phosphomimetic, participates in cell adhesion but does not interact with ankyrin or modulate I_Na_, suggesting that Y181 is an important regulatory point for cytoskeletal association and channel modulation (Malhotra et al., 2002). In CMs, tyrosine-phosphorylated Na_V_-β1 and non-phosphorylated Na_V_-β1 are differentially localized to subcellular domains where they interact with specific cytoskeletal and signaling proteins (Malhotra et al., 2004). At the T-tubules, non-phosphorylated Na_V_-β1 interacts with TTX-S Na_V_s and ankyrin-B (Figure 2) (Malhotra et al., 2004). In contrast, tyrosine-phosphorylated Na_V_-β1 is localized to the ID where it interacts with Na_v_1.5 and N-cadherin (Figures 3B,C) (Malhotra et al., 2004). We do not yet know whether phosphorylation targets Na_V_-β1 to specialized subcellular regions or whether Na_V_-β1 is differentially phosphorylated upon arrival. Phosphorylation may be a signaling mechanism by which cells regulate the density and localization of Na_V_-β1, and by association Na_v_-αs, to specific subcellular domains. In summary, Na_V_-β1 subunits serve as critical links between the extracellular and intracellular signaling environments of cells through ion channel modulation as well as cell–cell adhesion.

## Na_V_-β1 modulates potassium channels

Na_V_-β1 can interact with and modulate voltage-gated potassium channels (K_v_s) in addition to Na_V_s. K_v_-α subunits assemble as tetramers that normally associate with modulatory Kv-β subunits (Snyders, 1999). The K_v_4.x subfamily of channels express rapidly activating, inactivating, and recovering cardiac transient outward currents (I_to_) (Snyders, 1999). Co-expression of Na_V_-β1 with K_v_4.3 results in a ~four-fold increase in I_to_ density (Deschênes and Tomaselli, 2002). Additionally, Na_V_-β1 alters the voltage-dependence and kinetics of channel gating compared to K_v_4.3 expressed alone (Deschênes and Tomaselli, 2002). Importantly, Na_V_-β1 associates with K_v_4.2 and enhances its surface expression (Marionneau et al., 2012). Whole-cell voltage-clamp recordings obtained from cells expressing K_v_4.2 with Na_V_-β1 resulted in higher I_to_ densities compared to K_v_4.2 alone (Marionneau et al., 2012). Na_V_-β1 can also interact with and modulate K_v_1 (K_v_1.1, K_v_1.2, K_v_1.3, or K_v_1.6) and K_v_7 (K_v_7.2) channels (Nguyen et al., 2012). Lastly, Na_V_-β1B can also associate with K_v_4.3, resulting in increased I_to_ (Hu et al., 2012). Thus, K_v_ currents can be modulated by Na_V_-β subunits, at least in heterologous expression systems. Transfection of neonatal rat ventricular myocytes with siRNA targeting Na_V_-β1 significantly reduced the expression of K_v_4.x protein and reduced both I_Na_ and I_to_ (Deschênes et al., 2008), suggesting that Na_V_-β1 can modulate K_v_ currents in the heart *in vivo*.

The inward rectifier current I_K1_, expressed by Kir2.1, is critical for setting the resting membrane potential and modulating the late-phase of repolarization and AP duration in CMs (Nerbonne and Kass, 2005). Similar to Na_v_1.5, Kir2.x channels contain a C-terminal PDZ-binding domain which mediates interaction with SAP97 and syntrophin (Matamoros et al., 2016). It is thought that Kir2.x channels associate in microdomains that include caveolin 3, Na_v_1.5, SAP97, and syntrophin (Vaidyanathan et al., 2013). Na_v_1.5 interacts with α1-syntrophin via an internal N-terminal PDZ-like binding domain in addition to the C-terminal PDZ-binding domains (Matamoros et al., 2016). Importantly, Na_v_1.5-β1 co-expression increases Kir2.1 and Kir2.2, but not Kir2.3, currents, again suggesting that these channels are functionally linked and that Na_v_-β1 is critical to the formation of multi-ion channel complexes.

## *In vivo* roles of *Scn1b*

Animal models have been instrumental in understanding the role of *Scn1b* in cardiac excitability. *Scn1b* deletion in mice results in severe seizures, ventricular arrhythmias, and sudden death prior to weaning (Chen, 2004). *Scn1b* null ventricular CMs exhibit prolonged AP repolarization, increased *Scn5a*/Na_v_1.5 gene and protein expression, increased *Scn3a* expression, increased transient and persistent I_Na_ density, and prolonged QT and RR intervals (Lopez-Santiago et al., 2007). In agreement with an adhesive role for β1, cytoskeletal disruption in CMs also results in increased persistent I_Na_ (Undrovinas et al., 1995). Consistent with this, ventricular CMs isolated from cardiac-specific *Scn1b* null mice have increased I_Na_ density, increased susceptibility to polymorphic ventricular arrhythmias, and altered intracellular calcium handling that is TTX-S (Lin et al., 2015). These data indicate that loss of *Scn1b* expression is arrhythmogenic, mediated by altered ion channel gene and protein expression, I_Na_, I_K_, and calcium handling. Cardiac specific *Scn1b* deletion increases the duration of calcium signaling, resulting in delayed afterdepolarizations (Lin et al., 2015). It will be interesting to determine if expression of disease-associated *SCN1B* variants leads to dysfunctional ryanodine receptor signaling, which can also result in altered levels of intracellular calcium and the generation of arrhythmias (Bers, 2008; Fearnley et al., 2011; Glasscock, 2014).

The cardiac AP relies on the orchestration of multiple ion channels in concert. Na_V_-β1 is an important modulator of Na_v_-α as well as some K_V_- and Kir-α subunits. Na_V_ and K channels may be functionally linked through Na_V_-β1/β1B, and if so, defects in this mechanism may contribute to cardiac disease. It will be critical to determine the physiological effects of Na_V_-β1 interaction with other K channels, calcium channels, or other calcium-handling proteins at the T-tubules. It is intriguing to consider that Na_V_-β1 may serve as a central communication hub between sodium, potassium, and calcium channel families to coordinate depolarization, repolarization, and calcium signaling in CMs.

## *SCN1B* and human disease

*SCN1B* variants are implicated in a variety of inherited pathologies, including epileptic encephalopathy and cardiac arrhythmias (O'Malley and Isom, 2015) (Figure 1, Table 1). The epileptic encephalopathy Dravet syndrome is linked to heterozygous variants in *SCN1A* leading to haploinsufficiency in most patients, however, a subset of patients has *SCN1B* homozygous loss-of-function variants (Patino et al., 2009). The leading cause of mortality in Dravet syndrome is Sudden Unexpected Death in Epilepsy (SUDEP) (Nobili et al., 2011; Kalume, 2013; Devinsky et al., 2016). *SCN1B* variants are also linked to inherited cardiac arrhythmia syndromes that increase the risk of sudden death, including BrS (Hu et al., 2012), LQTS (Riuró et al., 2014), atrial arrhythmias (Watanabe et al., 2009), and SIDS (Hu et al., 2012). Diagnostic overlap between epilepsy and cardiac conduction disease can confound causative links between the two phenotypes (Ravindran et al., 2016). Cardiac conduction abnormalities can be poorly recognized in patients with epilepsy and vice versa (Zaidi et al., 2000). A retrospective electrocardiography study revealed that abnormal ventricular conduction was more common in SUDEP cases than in epileptic controls (Chyou et al., 2016). We propose that variants in *SCN1B*, including those linked to epilepsy, predispose patients to compromised cardiac electrical abnormalities. Thus, cardiovascular evaluation may be helpful in treating epileptic encephalopathy patients.

## Summary

Na_V_-β1 and -β1B are multifunctional molecules that associate with Na_v_ and K channels, cytoskeletal proteins, CAMs, and extracellular matrix molecules in the heart and brain. In addition, Na_V_-β1/β1B modulate multiple ionic currents, channel expression levels, and channel subcellular localization. Thus, it is not surprising that variants in *SCN1B* are linked to devastating cardiac and neurological diseases with a high risk of sudden death. In the field of cardiac physiology, important questions remain regarding specific cardiac Na_V_-β1 binding partners, potential effects of Na_V_-β1 on calcium-handling, the potential role of Na_V_-β1 in Na_v_1.5 dimerization, and the mechanism of phosphorylation events that affect Na_V_-β1 targeting to and association with subcellular domain specific signaling complexes at the ID, lateral membrane, and T-tubules. Understanding the functions of Na_V_-β1 within these protein complexes will help to elucidate underlying mechanisms of cardiac arrhythmias and associated sudden death, as well as lead to the discovery of novel biomarkers and therapeutic targets for human disease.

## Author contributions

All authors listed have made a substantial, direct and intellectual contribution to the work, and approved it for publication.

### Conflict of interest statement

The authors declare that the research was conducted in the absence of any commercial or financial relationships that could be construed as a potential conflict of interest.
